# Clinical Tools for Optimizing Therapeutic Decision-Making in Prostate Cancer: A Five-Year Retrospective Analysis

**DOI:** 10.3390/life14070838

**Published:** 2024-06-30

**Authors:** Silviu Constantin Latcu, Alin Adrian Cumpanas, Vlad Barbos, Victor-Bogdan Buciu, Marius Raica, Flavia Baderca, Pusa Nela Gaje, Raluca Amalia Ceausu, Cristina-Stefania Dumitru, Dorin Novacescu, Talida Georgiana Cut, Ligia Petrica

**Affiliations:** 1 Doctoral School, Victor Babes University of Medicine and Pharmacy Timisoara, E. Murgu Square, No. 2, 300041 Timisoara, Romania; silviu.latcu@umft.ro (S.C.L.); vlad.barbos@umft.ro (V.B.); victor.buciu@umft.ro (V.-B.B.); 2 Department XV, Discipline of Urology, Victor Babes University of Medicine and Pharmacy Timisoara, E. Murgu Square, No. 2, 300041 Timisoara, Romania; 3 Department II of Microscopic Morphology, Victor Babes University of Medicine and Pharmacy Timisoara, E. Murgu Square, No. 2, 300041 Timisoara, Romania; marius.raica@umft.ro (M.R.); baderca.flavia@umft.ro (F.B.); gaje.nela@umft.ro (P.N.G.); ra.ceausu@umft.ro (R.A.C.); cristina-stefania.dumitru@umft.ro (C.-S.D.); novacescu.dorin@umft.ro (D.N.); 4 Angiogenesis Research Center, Victor Babes University of Medicine and Pharmacy Timisoara, E. Murgu Square, No. 2, 300041 Timisoara, Romania; 5 Department XIII, Discipline of Infectious Diseases, Victor Babes University of Medicine and Pharmacy Timisoara, E. Murgu Square, No. 2, 300041 Timisoara, Romania; talida.cut@umft.ro; 6 Center for Ethics in Human Genetic Identifications, Victor Babes University of Medicine and Pharmacy Timisoara, E. Murgu Square, No. 2, 300041 Timisoara, Romania; 7 Department of Internal Medicine II, Division of Nephrology, “Victor Babeș” University of Medicine and Pharmacy, Eftimie Murgu Square, No. 2, 300041 Timișoara, Romania; petrica.ligia@umft.ro; 8 Centre for Molecular Research in Nephrology and Vascular Disease, Faculty of Medicine, “Victor Babeș” University of Medicine and Pharmacy, Eftimie Murgu Square, No. 2, 300041 Timișoara, Romania

**Keywords:** radical prostatectomy, prostate cancer, surgical treatment, clinical predictive tools, risk stratification, mortality, diagnosis, histopathology, imaging, prognosis

## Abstract

The effective staging of prostate cancer is essential for optimizing treatment and predicting outcomes. This study assessed the correlation between detailed preoperative diagnostic scores and postoperative outcomes to evaluate the accuracy of cancer restaging and its impact on treatment decisions and prognosis after prostatectomy. This retrospective study analyzed 133 prostate cancer patients who underwent prostatectomies at “Pius Brinzeu” Clinical Emergency Hospital in Timisoara over five years. Preoperative Gleason scores increased significantly across risk categories, from an average of 6.21 in low-risk patients to 7.57 in high-risk patients. This trend continued postoperatively, with scores rising from 7.04 to 8.33, respectively. The average increase in Gleason scores from preoperative to postoperative assessments was most pronounced in high-risk patients, at 0.76. Significant changes in clinical staging included increases in NCCN risk, where high-risk patients showed a 30% increase, and ISUP grade, with a 26.7% increase in the high-risk category. Notably, nodal status changes were also significant in high-risk patients, showing a 23.3% increase. The incidence of MRI-detected adenopathy was notably higher in the high-risk group (50%). Furthermore, there were significant correlations between the preoperative CAPRA score and postoperative ISUP grade (r = 0.261) and the preoperative PIRADS score and postoperative ISUP grade (r = 0.306). Similar observations were made between the preoperative and postoperative Gleason scores (r = 0.286) and the number of positive fragments (r = 0.227) with the postoperative ISUP grading. Furthermore, the preoperative CAPRA score was significantly correlated (r = 0.261) with the postoperative ISUP grading. Preoperative MRI findings, which included assessments of adenopathy and seminal vesicle invasion, were also significantly correlated (r = 0.218) with the postoperative pathological findings. Additionally, a significant correlation was found between the preoperative PIRADS score and postoperative ISUP grade (r = 0.306). In forecasting the aggressiveness and staging of prostate cancer following surgery, preoperative PSA levels showed an AUC of 0.631; the preoperative Gleason score had an AUC adjusted to 0.582, and the number of positive biopsy fragments indicated an AUC of 0.566. These results highlight the necessity of accurate and comprehensive preoperative assessments to better predict disease progression and refine treatment strategies.

## 1. Introduction

Prostate cancer remains one of the most prevalent malignancies among men worldwide, posing significant challenges in diagnosis, management, and follow-up [1,2]. The standard approach after diagnosis typically involves a combination of clinical evaluation, imaging, and histopathological examination to guide therapeutic decisions [3,4]. Among the surgical options, prostatectomy is the most common intervention with curative intention [5]. However, the postoperative management and restaging of prostate cancer are critical to determining residual disease, the likelihood of recurrence, and the need for additional treatment [6,7].

The importance of accurate restaging in prostate cancer cannot be overstated, as it directly influences subsequent therapeutic strategies and prognostic assessments [8]. Current methods for restaging typically involve a combination of prostate-specific antigen (PSA) levels, imaging studies such as MRI or CT scans, and sometimes bone scans, depending on the initial stage and grade of the tumor [9,10]. Moreover, histopathological analysis of the prostatectomy specimen offers detailed insights into the tumor pathology, providing valuable information on tumor margins, lymph node involvement, and other microscopic features [11]. Failing to accurately determine the extent of disease immediately following surgery can lead to either over-treatment or under-treatment, each carrying its own risks and implications for patient quality of life [12,13].

Considering these facts, the preoperative phase also plays an important role, as it sets the baseline for comparing postoperative outcomes [14]. The initial staging, which involves clinical evaluations, imaging assessments, and histopathological scoring, must be robust and thorough to ensure that the baseline is accurate and comprehensive. Nevertheless, postsurgical restaging is essential to determine the future management of the disease. The objectives of this retrospective analysis are to assess the effectiveness of integrating detailed preoperative diagnostic scores with postoperative outcomes and to determine the impact of such an integration on the accuracy of cancer restaging, treatment decisions, and prognosis after prostatectomy.

## 2. Materials and Methods

### 2.1. Research Design and Ethical Considerations

This retrospective analysis was designed to evaluate the prostatectomies performed for prostate cancer at a single university center in the western Romanian region, at the Department of Urology of “Pius Brinzeu” Clinical Emergency Hospital from Timisoara, during a 5-year period.

Adhering to strict ethical standards, the research protocol was reviewed and approved by the Institutional Review Board of the medical center, which operates under provisions aligned with the EU Good Clinical Practice Directives 2005/28/EC, the ICH guidelines for ethical research, and the principles outlined in the Declaration of Helsinki.

Informed consent was retrospectively collected from all participants whose data were included in the study from their medical paper records. All researchers involved in the study were trained in data handling and privacy protection, ensuring compliance with the ethical standards throughout the research process.

### 2.2. Inclusion Criteria

The initial phase of the selection process involved a thorough screening of the hospital’s electronic health records system and the paper medical records using the primary diagnosis of prostate cancer as the search criterion. This was further refined through a detailed review of preoperative histopathological reports, imaging results (e.g., MRI, CT scans), and clinical scores (e.g., PSA levels).

The inclusion criteria were as follows: adult male patients aged 40 years or older at the time of diagnosis who underwent prostatectomy, had complete preoperative and postoperative clinical, imaging, and histopathological data available, and demonstrated a willingness to have their medical data used for research purposes. Consent for participation was part of the initial treatment consent, where permissible.

The exclusion criteria included patients with prior prostate surgeries, those who had received oncological therapy (e.g., chemotherapy or radiation therapy) before the prostatectomy, or those lacking complete preoperative or postoperative data. Additional exclusion criteria were applied to patients who did not have a clear pathological confirmation of prostate cancer, those with metastatic disease at the time of diagnosis, and individuals who withdrew consent for their data to be used in research. Cases with inconsistent data between EHR entries and physical records were also excluded to maintain the integrity of the study. From the total number of 237 prostatectomies performed during the five-year study period, 133 cases were eligible for inclusion in the final analysis based on the established inclusion criteria.

### 2.3. Variables and Definitions

In this study, data were stratified into comparison groups based on clinically significant variables to assess and compare the outcomes across different risk levels and diagnostic classifications. Prostate Imaging Reporting and Data System (PIRADS) scores were categorized into PIRADS 1–2, 3, 4, and 5, facilitating the analysis of groups with escalating suspicion of clinically significant prostate cancer [15]. The D’Amico Risk Classification system was utilized to segment patients into low, intermediate, and high-risk groups based on the initial PSA levels, Gleason scores, and clinical T stage, providing a traditional framework for prostate cancer prognosis and treatment pathway decisions [16]. Additionally, the International Society of Urological Pathology (ISUP) Grade Group was applied, refining patient stratification from Groups 1 through 5 based on a detailed assessment of histopathological findings [17]. For the clinical T stage (cT), data were divided into early-stage (cT1–2) and advanced-stage (cT3–4) groups, further detailed by each specific stage (cT1, cT2, cT3, and cT4) to tailor the analysis to the extent of primary tumor development. Finally, the Cancer of the Prostate Risk Assessment (CAPRA) score was used to classify participants into three risk categories: low (0–2), intermediate (3–5), and high (6–10), incorporating a composite of clinical factors for a nuanced longitudinal risk assessment and comparison of treatment outcomes [18].

### 2.4. Statistical Analysis

Data management and analysis were performed using SPSS version 26.0 (SPSS Inc., Chicago, IL, USA). Continuous variables were summarized as mean ± standard deviation (SD), and categorical variables as frequencies and percentages. Statistical tests included ANOVA for continuous variables and Chi-square for categorical data. Pearson’s rho, Spearman’s rank correlation, and ROC curve analyses were utilized to assess the predictive accuracy of preoperative factors on postoperative outcomes, with a significance threshold set at a *p*-value of less than 0.05. All results were thoroughly validated for accuracy and reliability.

## 3. Results

A total of 28 patients were identified as low risk based on the CAPRA score, as well as 75 with an intermediate risk, and 30 with a high risk, respectively. Age among the groups showed no significant difference, ranging between 47 and 79 years. The PSA levels, however, varied significantly with risk level, ranging from an average of 6.70 ng/mL in low-risk to 16.44 ng/mL in high-risk groups (*p*-value < 0.001). Prostate volume and density measurements, with averages around 40 to 43 cc and 0.18 to 0.43, respectively, did not demonstrate significant differences across the groups (*p*-values of 0.581 and 0.163, respectively).

Significant differences were noted in the identification and positioning of lesions. The frequency of identified lesions increased significantly from 25% in low-risk to 60% in high-risk groups (*p*-value = 0.022). The incidence of bilateral lesions followed a similar pattern, increasing with risk level (*p*-value = 0.022). Lesion dimensions also varied significantly, enlarging from an average of 0.94 cm in low-risk to 1.75 cm in high-risk groups (*p*-value < 0.001). Furthermore, MRI-detected adenopathy was significantly more prevalent in the high-risk group at 50%, compared to 21.4% in the low-risk group (*p*-value = 0.004). Changes in MRI seminal vesicle invasion did not reach statistical significance (*p*-value = 0.068), as presented in Table 1.

Clinical tumor staging varied across risk groups, with most low-risk patients classified as stage 2a (75.0%) and a notable progression to stages 1c and 2c in the intermediate and high-risk groups. However, the statistical analysis did not reveal significant differences (*p*-value = 0.252). Clinical node involvement indicated that a minimal number of patients in each risk group had node involvement: 7.1% in low risk, 18.7% in intermediate, and 6.7% in high risk. These differences were not statistically significant (*p*-value = 0.144).

The prostate biopsy pathology (PBP) Gleason scores showed a clear trend of increase with higher CAPRA scores: 6.21 in low risk, 6.88 in intermediate risk, and 7.57 in high risk, with these differences reaching statistical significance (*p*-value < 0.001). The ISUP grading revealed significant differences (*p*-value < 0.001) among the risk groups. While most low-risk patients fell into the ISUP grades 1–2, a considerable shift was observed in the intermediate and high-risk groups, where higher grades (3 and 4–5) became prevalent.

The number of sampled fragments showed no significant difference across risk groups (*p*-value = 0.810). However, the number of positive fragments differed significantly (*p*-value < 0.001), escalating from an average of 2.32 in low risk to 7.90 in high risk. The Briganti score, which assesses the probability of lymph node invasion, also significantly increased with higher risk categories, from an average of 2.71 in low risk to 59.27 in high risk (*p*-value < 0.001). Finally, the positive/total ratio of sampled fragments and acinar growth pattern did not yield statistically significant differences (*p*-value = 0.151 for both), as presented in Table 2.

The interval from prostate biopsy to prostatectomy did not show a significant variation between groups, averaging around 98.71 days for low risk, 105.88 days for intermediate risk, and 93.73 days for high risk. The CPG grading revealed significant differences in postoperative staging, where 86.4% of low-risk patients were classified as CPG 1–2, compared to 53.3% of intermediate and none in the high-risk group. Conversely, 70% of high-risk patients fell into the CPG 4–5 category.

The NCCN risk categorization also showed marked differences, with 78.6% of low-risk patients falling into NCCN 1–2, but this figure dropped to 6.7% in the intermediate group and was null in high-risk patients. Most high-risk patients were categorized into NCCN 5–6 (66.7%), suggesting a progression to the higher risk categories postoperatively. The Radical Prostatectomy (RRP) Gleason scores remained consistent with preoperative risk assessments; low-risk patients had an average score of 7.04, which increased to 8.33 in the high-risk group.

Perineural invasion and positive surgical margins were commonly observed across all risk categories, with rates exceeding 85% and 30%, respectively. The International Society of Urological Pathology (ISUP) grading based on RRP showed that 89.3% of low-risk patients were within ISUP 1–2, compared to only 8% for high-risk patients. High-risk patients predominantly fell within ISUP 4–5 (46.6%). The American Joint Committee on Cancer (AJCC) staging based on RRP also highlighted the severity of disease progression in high-risk groups, where 53.3% were categorized as stage 4, compared to none in the low-risk group (Table 3).

The preoperative prostate biopsy pathology (PBP) Gleason scores were significantly different across the risk categories, increasing from a mean of 6.21 in low-risk to 7.57 in high-risk patients (*p* < 0.001). This trend persisted in the postoperative radical prostatectomy (RRP) Gleason scores, which also showed significant differences, escalating from 7.04 in the low-risk group to 8.33 in the high-risk group (*p* < 0.001). The differences between preoperative and postoperative Gleason scores within each group were statistically significant, with the mean differences reflecting varying degrees of underestimation by initial biopsy compared to the surgical specimen, notably higher in the high-risk group (*p* = 0.037).

Progression in pathological tumor (pT) staging postoperatively was observed but did not reach statistical significance across groups (*p* = 0.164), suggesting variable but not uniformly significant progression in tumor extent after surgery. The increase in NCCN risk categories postoperatively showed a significant variation (*p* = 0.010), with the high-risk group experiencing the most substantial increase in risk classification, indicating a potential underestimation of disease severity at initial staging.

Similarly, the ISUP grade increases were significant (*p* = 0.008), with a more pronounced grade progression in high-risk patients, supporting the correlation with more aggressive disease characteristics uncovered during surgery. Changes in nodal status (N+), indicating the presence of cancer in the lymph nodes, also varied significantly (*p* = 0.042). The change in positive surgical margins and the transition to higher T stages (T3 or T4) were analyzed, but these did not demonstrate statistically significant differences (*p* = 0.270 and *p* = 0.519, respectively), as seen in Table 4.

The analysis revealed that the prostate-specific antigen (PSA) levels had a low correlation (coefficient = 0.087) with postoperative Gleason scores, and this relationship was not statistically significant (*p*-value = 0.319). Similarly, the number of positive nodes showed a modest correlation (coefficient = 0.168) with postoperative Gleason scores (*p*-value = 0.054).

A stronger and statistically significant correlation was observed between the preoperative and postoperative Gleason scores (coefficient = 0.286, *p*-value = 0.001), suggesting that initial Gleason scoring is a reliable predictor of postoperative histological outcomes. The number of positive fragments also demonstrated a significant correlation (coefficient = 0.227) with the postoperative ISUP grading (*p*-value = 0.038), supporting the prognostic value of the number of positive biopsy fragments in determining the aggressiveness of prostate cancer as defined by postoperative ISUP grades.

Furthermore, the preoperative CAPRA score was significantly correlated (coefficient = 0.261) with the postoperative ISUP grading (*p*-value = 0.009), underscoring the effectiveness of the CAPRA scoring system in predicting postoperative pathological outcomes. Preoperative MRI findings, which included assessments of adenopathy and seminal vesicle invasion, were also significantly correlated (coefficient = 0.218) with postoperative pathological findings (*p*-value = 0.042). Additionally, a significant correlation was found between the preoperative PIRADS score and postoperative ISUP grade (coefficient = 0.306, *p*-value = 0.001), indicating a strong predictive relationship between PIRADS scoring and postoperative tumor grade (Table 5).

**Figure 1:**
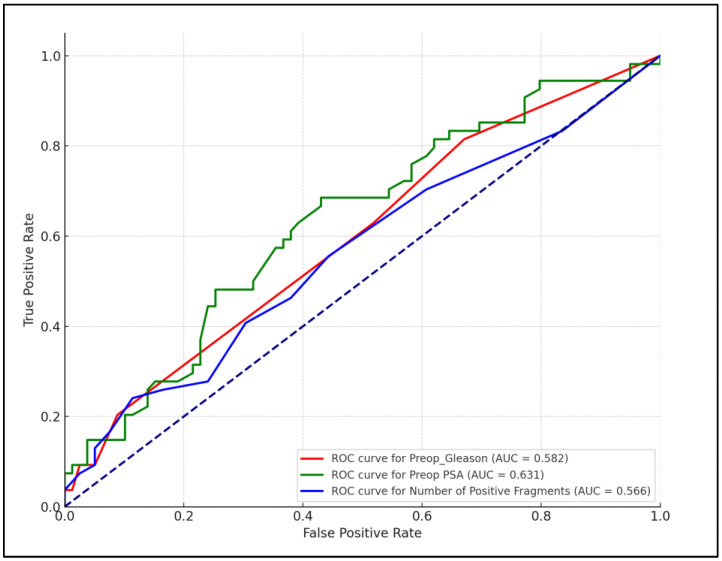
ROC analysis for preoperative factors predicting postoperative prostate cancer stage.

Figure 1 presents the receiver operating characteristic (ROC) curves for several preoperative factors and their predictive capabilities regarding postoperative prostate cancer staging. The ROC curves demonstrate the performance of preoperative PSA levels, Gleason scores, and the number of positive biopsy fragments in forecasting the aggressiveness and staging of prostate cancer following surgery. The preoperative PSA levels showed an AUC of 0.631, indicating moderate predictive power; the preoperative Gleason score had an AUC adjusted to 0.582, reflecting a fair prediction capability; and the number of positive biopsy fragments indicated an AUC of 0.566, suggesting limited but notable predictive relevance.

## 4. Discussion

### 4.1. Important Findings and Literature Review

This study elucidates the nuanced relationship between preoperative diagnostic tools and postoperative outcomes, with a particular focus on the predictability of CAPRA scores and MRI findings in relation to postoperative ISUP grades. The data indicate a significant correlation between the CAPRA scores and the postoperative ISUP grades, highlighting the utility of CAPRA in preoperative risk stratification. Patients with higher CAPRA scores tended to have higher ISUP grades postoperatively, suggesting that the CAPRA scores effectively capture the biologic aggressiveness of prostate cancer, which is crucial for tailoring treatment strategies.

Even though the initial risk assessment in our study was conducted based on the CAPRA score, it was observed that at the later MRI findings, the lymph node involvement and seminal vesicle invasion differed significantly from these initial assessments for some patients categorized as low risk, with a proportion of 21.4% being categorized as low but later identified with a higher risk based on these observations. Additionally, there was a significant 30% NCCN postoperative risk increase among patients initially identified as high risk and a 26.7% increase in ISUP grade. These discrepancies underscore the importance of considering more comprehensive diagnostic tools in the staging of prostate cancer. Our findings point to the necessity of restaging in certain cases, where MRI and other advanced imaging techniques reveal a more accurate picture of the disease’s extent and severity. Therefore, this study advocates for the integration of imaging findings into the prostate cancer staging process to enhance the accuracy of risk assessment and treatment planning. The study’s findings strengthen the argument for the integration of detailed MRI assessments into standard preoperative evaluations to improve the specificity of treatment approaches.

Additionally, the correlation analysis extended to the preoperative PIRADS scores and postoperative ISUP grades provides further clinical utility by confirming the effectiveness of PIRADS scores in predicting postoperative histological outcomes. This alignment suggests that higher PIRADS scores, which indicate a higher probability of clinically significant prostate cancer, are consistent with more advanced disease stages, as confirmed by postoperative pathology.

These correlations between preoperative assessments and postoperative findings emphasize the critical role of comprehensive preoperative evaluations in enhancing the predictive accuracy of prostate cancer staging. The clinical utility of these findings lies in their potential to refine patient counseling, guide surgical decision-making, and optimize personalized treatment plans, ultimately aiming to improve patient outcomes in prostate cancer care.

Other studies presented offer critical insights into prostate cancer restaging using advanced imaging techniques, specifically [68Ga]Ga-PSMA-11 PET/CT and [11C]choline PET/CT, each within the context of biochemical recurrence. Fourquet et al. [8] demonstrate that [68Ga]Ga-PSMA-11 PET/CT had both a sensitivity and specificity of 70%, leading to changes in patient management in 68% of cases, with treatment effectiveness reaching 78% overall, and 89% when guided by [68Ga]Ga-PSMA-11 PET/CT. In contrast, Giovacchini and Breeuwsma [19] report a variable positive detection rate of [11C]choline PET/CT ranging between 40% and 70%, heavily influenced by PSA levels and patient characteristics such as PSA kinetics and hormonal resistance. Both studies underscore the significant role of PET/CT in enhancing diagnostic accuracy and influencing treatment pathways.

Similarly, Castellucci et al. [20] assessed the detection rate of [11C]choline PET/CT in patients scheduled for salvage radiation therapy, finding a positivity rate of 28.4% among 605 patients, with a significant association between PSA levels, PSA doubling time, and scan positivity. Notably, they found that ongoing androgen deprivation therapy also influenced the PET/CT outcomes, with optimal cutoff values for PSA and PSA doubling time enhancing scan prediction accuracy. Conversely, von Eyben et al. [21] conducted a meta-analysis involving 1216 patients using [68Ga]Ga-PSMA-11 PET/CT, developing a risk model based on prescan PSA levels that markedly predicted five-year overall survival. They reported higher survival rates in patients with more extensive disease detected on PET/CT. Moreover, Kabasakal et al. [22] focused on the diagnostic sensitivity and specificity of PSMA PET/CT in patients with low PSA levels (under 5 ng/mL), finding that the positivity rate of PET scans increased with rising PSA levels, achieving a sensitivity of 76.47% and a specificity of 91.67%. This study also highlighted the effectiveness of PSMA PET/CT in detecting recurrent disease even at very low PSA levels, suggesting its potential in early restaging and influencing treatment decisions.

Aside from PET/CT, another study [23] utilized DCE-MRI to assess the local failures before salvage radiotherapy, revealing that 28.4% of their 605 patients showed positive scans. Notably, patients with lesions at the vesico-urethral anastomosis had a significantly higher 4-year bNED rate of 94.6% compared to those with lesions elsewhere or larger volumes. Cordero da Luz et al., analyzing data from over 404,210 men, focused on the prognostic impact of lymph node involvement detected during prostatectomy [24]. Their findings underscored that patients with 1+, 2+, and >2+ lymph nodes affected had redefined staging criteria that provided a more accurate prognosis, with a specific survival C-index improvement from 0.892 to 0.908 when using their new staging criteria. These studies emphasize the critical role of precise loco-regional staging through either DCE-MRI or surgical assessment in refining therapeutic strategies and improving prognostic accuracy in prostate cancer management.

Building upon the insights regarding the value of precise staging via DCE-MRI and surgical assessments, the subsequent exploration by Murray et al. [25] pivots towards the comparative efficacy of predictive scoring systems in post-prostatectomy prostate cancer management. This shift in focus from anatomical to biochemical markers, such as the CAPRA-S score versus minimal residual disease (MRD) markers, illustrates the evolving landscape of diagnostic strategies aiming to refine prognosis and therapeutic decision-making. The incorporation of these diverse diagnostic tools emphasizes an integrated approach that seeks to combine anatomical, biochemical, and molecular insights to enhance the accuracy and personalized nature of prostate cancer treatment. They demonstrated that the CAPRA-S score, when compared to MRD markers, was less effective in differentiating patient outcomes; MRD identified groups with significantly different biochemical failure-free survival (BFFS): Group A (no MRD) showed a 10-year BFFS of 95%, while Group C (CPC positive) had only 27% BFFS. In contrast, Chang et al. [26] found that incorporating imaging-based T staging into the CAPRA score affected the staging of 377 out of 2222 patients (17%), but both imaging-based and digital rectal examination-based CAPRA scores similarly predicted biochemical recurrence (HR 1.52 vs. HR 1.54). These findings suggest that while traditional CAPRA scoring remains a robust predictor of recurrence across different staging modalities, the integration of biological markers like MRD could provide superior specificity in predicting long-term outcomes, especially in identifying patients at varying risk levels post-surgery.

Shifting from the nuanced improvements in prostate cancer prognosis through molecular and imaging assessments, the studies by Yang et al. [27,28] take a broader leap into the realm of oncological treatment advancements. This research introduces innovative nanoantidote strategies aimed at mitigating the harsh systemic effects of chemotherapy without undermining its effectiveness against cancer, illustrating a pivotal shift towards enhancing patient care through technological integration in treatment protocols. This approach, which involves pre-administering nanoantidotes that selectively bind to cisplatin in target organs, optimizes the therapeutic window by preserving the drug’s effectiveness while minimizing adverse effects. This method contrasts with traditional antidotes that often reduce both toxicity and therapeutic efficacy. The study’s results are promising, indicating that such nanoantidotes can significantly enhance patient tolerance to potent chemotherapeutic agents, which could lead to broader clinical applications and improved patient outcomes. Meanwhile, the research by Cheng et al. [29] on MT1G in prostate cancer sheds light on the potential of ferroptosis-related genes as biomarkers for immunotherapy sensitivity. The significant downregulation of MT1G in prostate cancer tissues, associated with poor prognosis, and its role in regulating the tumor microenvironment and immune response highlight its utility not only as a prognostic marker but also as a potential therapeutic target. The ability of MT1G to modulate immune infiltration and enhance the effectiveness of immune checkpoint inhibitors could lead to novel treatment strategies in prostate cancer, particularly for patients resistant to conventional therapies. Both studies illustrate the critical intersections of nanotechnology and molecular biology in advancing personalized medicine and improving therapeutic outcomes in oncology.

This study demonstrated that higher preoperative Gleason and CAPRA scores correlated significantly with increased postoperative ISUP grades, particularly highlighting a pronounced progression in high-risk prostate cancer patients. Clinically, these correlations validate the use of these preoperative metrics to anticipate aggressive disease behavior and guide decision-making for more personalized surgical approaches. The observed trends in diagnostic scores offer clinicians a predictive tool for refining treatment plans and potentially improving outcomes by aligning therapeutic strategies more closely with individual patient risk profiles. Significantly, the correlation between preoperative MRI findings and postoperative pathology further reinforces the importance of advanced imaging in staging and treatment planning, making a compelling case for its broader implementation in clinical practice.

### 4.2. Study Limitations and Future Perspectives

This study faces several limitations that must be acknowledged. Firstly, its retrospective nature and the single-center design may limit the generalizability of the findings. The sample size, although adequate for statistical analysis, represents a relatively small cohort from a specific geographical area, which might not fully capture the diversity seen in broader populations. Additionally, inherent biases associated with retrospective data collection and reliance on historical medical records could influence the accuracy and completeness of the data. Moreover, while significant correlations were found among certain preoperative and postoperative parameters, the causative mechanisms behind these associations remain to be explored in detail. Future studies could benefit from a multi-center design, larger sample sizes, and prospective data collection to validate these findings. Incorporating genetic markers and molecular profiling into the preoperative assessment could also enhance predictive accuracy and personalized treatment planning in prostate cancer management.

## 5. Conclusions

The findings from this study highlight the significant role of detailed preoperative diagnostic evaluations in predicting postoperative outcomes in prostate cancer patients. Notably, the study established strong correlations between preoperative factors such as CAPRA scores, MRI findings, and PIRADS scores with postoperative ISUP grades. These correlations suggest that comprehensive preoperative assessments can effectively predict the pathological stage of prostate cancer, which is important for optimizing treatment decisions and improving prognostic accuracy. The study also underscores the potential underestimation of disease severity by traditional staging methods, advocating for the integration of advanced diagnostic tools in routine clinical practice. These results provide a valuable foundation for enhancing prostate cancer staging protocols and tailoring treatment strategies to individual patient profiles, ultimately aiming to improve clinical outcomes in prostate cancer care.

## Figures and Tables

**Table 1:** Characterization of initial findings in patients with prostate cancer by CAPRA score before prostatectomy.

Variables	Low Risk (*n* = 28)	Intermediate Risk (*n* = 75)	High Risk (*n* = 30)	*p*-Value
Age, years (mean ± SD)	63.18 ± 4.37	64.89 ± 8.82	63.43 ± 6.44	0.994
PSA (mean ± SD)	6.70 ± 2.22	11.13 ± 8.77	16.44 ± 8.23	<0.001
Volume (mean ± SD)	40.61 ± 14.76	43.43 ± 19.27	40.77 ± 12.35	0.581
Density (mean ± SD)	0.18 ± 0.08	0.31 ± 0.43	0.43 ± 0.24	0.163
TR				0.709
0	6 (21.4%)	14 (18.7%)	4 (13.3%)	
1	22 (78.6%)	61 (81.3%)	26 (86.7%)	
PIRADS				0.143
1–2	18 (64.3%)	41 (54.7%)	15 (50.0%)	
3–4	8 (28.6%)	30 (40.0%)	9 (30.0%)	
5	2 (7.1%)	4 (5.3%)	6 (20.0%)	
Identified lesions				0.022
0	21 (75.0%)	46 (61.3%)	12 (40.0%)	
1	7 (25.0%)	29 (38.7%)	18 (60.0%)	
Position				0.022
Unilateral	21 (75.0%)	46 (61.3%)	12 (40.0%)	
Bilateral	7 (25.0%)	29 (38.7%)	18 (60.0%)	
Dimension/Size (mean ± SD)	0.94 ± 0.77	1.34 ± 0.98	1.75 ± 1.23	<0.001
MRI adenopathy	6 (21.4%)	14 (18.7%)	15 (50.0%)	0.004
MRI seminal vesicle invasion	6 (21.4%)	12 (16.0%)	11 (36.7%)	0.068

Risk groups were assessed based on the CAPRA (Cancer of the Prostate Risk Assessment) score. SD—standard deviation; PIRADS—Prostate Imaging Reporting and Data System.

**Table 2:** Pathological features in patients with prostate cancer by CAPRA score before prostatectomy.

Variables	Low Risk (*n* = 28)	Intermediate Risk (*n* = 75)	High Risk (*n* = 30)	*p*-Value
cT	2a: 21 (75.0%), 1c: 6 (21.4%), 2c: 1 (3.6%)	2a: 52 (69.3%), 1c: 14 (18.7%), 2c: 9 (12.0%)	2a: 19 (63.3%), 3b: 4 (13.3%), 1c: 7 (23.3%)	0.252
cN (0)	2 (7.1%)	14 (18.7%)	2 (6.7%)	0.144
PBP Gleason score (combined)	6.21 ± 0.42	6.88 ± 0.49	7.57 ± 0.73	<0.001
ISUP grade				<0.001
1–2	22 (78.6%) 6 (21.4%)	14 (18.7%) 47 (62.7%)	5 (16.7%) 13 (43.3%)	
3	0 (0.0%)	10 (13.3%)	13 (43.3%)	
4–5	0 (0.0%)	4 (5.3%)	12 (40.0%)	
Number of sampled fragments (mean ± SD)	11.39 ± 1.99	11.61 ± 2.87	12.07 ± 1.86	0.810
Number of positive fragments (mean ± SD)	2.32 ± 1.28	4.29 ± 3.14	7.90 ± 3.60	<0.001
Positive/Total ratio				0.151
Grade 1 fragments	27 (96.4%)	75 (100.0%)	30 (100.0%)	
Grade 2 fragments	1 (3.6%)	0 (0.0%)	0 (0.0%)	
Briganti score (mean ± SD)	2.71 ± 2.02	14.45 ± 18.89	59.27 ± 26.54	<0.001
Acinar growth pattern	27 (96.4%)	75 (100.0%)	30 (100.0%)	0.151

Risk groups were assessed based on the CAPRA (Cancer of the Prostate Risk Assessment) score. SD—standard deviation; cT—clinical tumor stage; cN—clinical node involvement stage; ISUP—International Society of Urological Pathology.

**Table 3:** Pathological features after prostatectomy characterized by CAPRA score.

Variables	Low Risk (*n* = 28)	Intermediate Risk (*n* = 75)	High Risk (*n* = 30)	*p*-Value
Interval from PBP to prostatectomy	98.71 ± 49.53	105.88 ± 57.87	93.73 ± 46.89	0.608
CPG				<0.001
1–2	27 (86.4%)	37 (53.3%)	0 (0.0%)	
3	1 (3.6%)	28 (37.3%)	9 (30.0%)	
4–5	0 (0.0%)	7 (9.3%)	21 (70.0%)	
NCCN risk				<0.001
1–2	22 (78.6%)	5 (6.7%)	0 (0.0%)	
3–4	5 (17.9%)	63 (84.0%)	10 (33.3%)	
5–6	0 (0.0%)	7 (9.3%)	20 (66.7%)	
pT	12 (42.9%) 11 (39.3%)	31 (41.3%) 29 (38.7%)	13 (43.3%) 12 (40.0%)	0.978
RRP Gleason score (combined)	7.04 ± 0.84	7.33 ± 0.86	8.33 ± 0.99	<0.001
Perineural invasion	24 (85.7%)	66 (88.0%)	26 (86.7%)	0.948
Positive surgical margins	11 (39.3%)	32 (42.7%)	9 (30.0%)	0.485
ISUP grade based on RRP				<0.001
1–2	25 (89.3%)	52 (69.3%)	6 (8.0%)	
3	2 (7.1%)	20 (26.7%)	12 (40.0%)	
4–5	1 (3.6%)	3 (4.0%)	14 (46.6%)	
AJCC staging based on RRP				<0.001
1	5 (17.9%)	2 (2.7%)	0 (0.0%)	
2	12 (42.9%)	30 (40.0%)	4 (13.3%)	
3	11 (39.3%)	37 (49.3%)	10 (33.3%)	
4	0 (0.0%)	6 (8.0%)	16 (53.3%)	

Risk groups were assessed based on the CAPRA (Cancer of the Prostate Risk Assessment) score. SD—standard deviation; cT—clinical tumor stage; cN—clinical node involvement stage; PBP—prostate biopsy pathology; ISUP—International Society of Urological Pathology; CPG—Cambridge Prognostic Group; NCCN—National Comprehensive Cancer Network; pT—pathological stage; RRP—radical prostatectomy; AJCC—American Joint Committee on Cancer.

**Table 4:** Restaging outcomes.

Variables	Low Risk (*n* = 28)	Intermediate Risk (*n* = 75)	High Risk (*n* = 30)	*p*-Value
Preoperative (PBP) Gleason	6.21 ± 0.42	6.88 ± 0.49	7.57 ± 0.73	<0.001
Postoperative (RRP) Gleason	7.04 ± 0.84	7.33 ± 0.86	8.33 ± 0.99	<0.001
Difference	0.83 ± 0.94	0.45 ± 0.99	0.76 ± 1.23	0.037
Progression of pT staging	3.6%	16.0%	20.0%	0.164
NCCN risk increase	7.1%	9.3%	30.0%	0.010
ISUP grade increase	3.6%	8.0%	26.7%	0.008
Change in T stage (T3 or T4)	7.1%	14.7%	16.7%	0.519
Change in nodal status (N+)	3.6%	9.3%	23.3%	0.042
Change in positive margins	10.7%	12.0%	23.3%	0.270

Risk groups were assessed based on the CAPRA (Cancer of the Prostate Risk Assessment) score. SD—standard deviation; cT—clinical tumor stage; cN—clinical node involvement stage; PBP—prostate biopsy pathology; ISUP—International Society of Urological Pathology; CPG—Cambridge Prognostic Group; NCCN—National Comprehensive Cancer Network; pT—pathological stage; RRP—radical prostatectomy; AJCC—American Joint Committee on Cancer.

**Table 5:** Correlation analysis.

Variables	Correlation Coefficient	*p*-Value
PSA and postoperative Gleason score	0.087	0.319
Number of positive nodes and postoperative Gleason score	0.168	0.054
Preoperative Gleason score and postoperative Gleason score	0.286	0.001
Number of positive fragments and postoperative ISUP	0.227	0.038
Preoperative CAPRA score and postoperative ISUP	0.261	0.009
Preoperative MRI findings (adenopathy/seminal vesicle invasion) and postoperative pathological findings	0.218	0.042
Preoperative PIRADS score and postoperative ISUP	0.306	0.001

ISUP—International Society of Urological Pathology; PSA—prostate-specific antigen; CAPRA—Cancer of the Prostate Risk Assessment; PIRADS—Prostate Imaging Reporting and Data System.

## Data Availability

The data presented in this study are available on request from the corresponding author.
